# Routes for COVID-19 importation in Brazil

**DOI:** 10.1093/jtm/taaa042

**Published:** 2020-03-23

**Authors:** Darlan Da S Candido, Alexander Watts, Leandro Abade, Moritz U G Kraemer, Oliver G Pybus, Julio Croda, Wanderson de Oliveira, Kamran Khan, Ester C Sabino, Nuno R Faria

**Affiliations:** 1 Department of Zoology, University of Oxford, Oxford, UK; 2 Li Ka Shing Knowledge Institute, St. Michael’s Hospital, Toronto, ON, Canada; 3 Division of Infectious Diseases, Department of Medicine, University of Toronto, Toronto, ON, Canada; 4 Harvard Medical School, Harvard University, Boston, MA, USA; 5 Computational Epidemiology Group, Boston Children’s Hospital, Boston, MA, USA; 6 Department of Pathobiology and Population Sciences, The Royal Veterinary College, London, UK; 7 Secretaria de Vigilância em Saúde, Coordenação Geral de Laboratórios de Saúde Pública, Ministério da Saúde, Brasília, Brazil; 8 Laboratório de Pesquisa em Ciências da Saúde, Universidade Federal da Grande Dourados, Dourados Mato Grosso do Sul, Brazil; 9 Fundação Oswaldo Cruz Campo Grande, Campo Grande, Brazil; 10 Instituto de Medicina Tropical, University of São Paulo, São Paulo, Brazil; 11 Department of Infectious Disease Epidemiology, School of Public Health, Imperial College London

## Abstract

The global outbreak caused by the severe acute respiratory syndrome coronavirus-2 (SARS-CoV-2) has been declared a pandemic by the World Health Organization. As the number of imported SARS-CoV-2 cases is on the rise in Brazil, we use incidence and historical air travel data to estimate the most important routes of importation into the country.

Severe acute respiratory syndrome coronavirus-2 (SARS-CoV2) was first detected in Wuhan, Hubei province, China, on 8 December 2019. SARS-CoV-2 infection can cause coronavirus disease (COVID-19) and can lead to acute respiratory syndrome, hospitalization and death.^1^ As of 12 March 2020, the global SARS-CoV-2 outbreak has been declared a pandemic, with 125 048 cases, and 4613 deaths have been notified by the World Health Organization (WHO) in 117 countries/territories or areas worldwide (who.int/emergencies/diseases/novel-coronavirus-2019/situation-reports). The first case in Latin America was confirmed on 26 February 2020, in the São Paulo metropolis, the most populous city in the Southern hemisphere (~11 million people, Instituto Brasileiro de Geografia e Estatística, www.ibge.gov.br). Self-declared travel history and subsequent genetic analyses confirmed that the first detected infection was acquired via importation of the virus from Northern Italy.^2^ Since then, Brazil has reported the largest number of cases in Latin America (*n* = 34, as of 10 March 2020). SARS-CoV-2 has been now detected in 7 (26%) of the 27 federal states of Brazil. So far, the transmission of SARS-CoV-2 appears to be primarily sporadic (85.3%, 29/34 are imported cases). Here, we analyze data on airline travellers to Brazil in 2019, who departed from countries that had reported local cases of COVID-19 transmission by 5 March 2020. This information provides insights into which Brazilian cities are most at risk for SARS-CoV-2 importation.

We used travel data on all air journeys that had a Brazilian city as their final destination during February and March 2019 as a proxy for flight density during the 2020 COVID-2019 outbreak (see Supplementary data). We focused on the data for 29 countries that had reported SARS-CoV-2 cases by 5 March 2020. We collated the total number of passengers flying to Brazilian airports during this period, country population size for 2019 from the United Nations World Population Prospects 2019 database, and the WHO-reported number of COVID-19 cases (as of 5 March 2020). We used these values to estimate the proportion of infected travellers potentially arriving in Brazilian cities from each country and for each route (additional information can be found in Supplementary data). No air passenger data from Iran and Portugal to Brazil were available for our analysis. 

Between February and March 2019, Brazil received 841 302 international passengers in a total of 84 cities across the country (Figure 1). São Paulo, the largest city in the country, was the final destination of nearly half (46.1%) of the passengers arriving to Brazil, followed by Rio de Janeiro (21%) and Belo Horizonte (4.1%). More than half of the international passengers started their journey in the USA (50.8%) followed by France (7.9%) and Italy (7.5%). The air-travel routes to airports in Brazil with most passengers were USA–São Paulo (23.3%), USA–Rio de Janeiro (9.8%) and Italy–São Paulo (3.4%).

**Figure 1:**
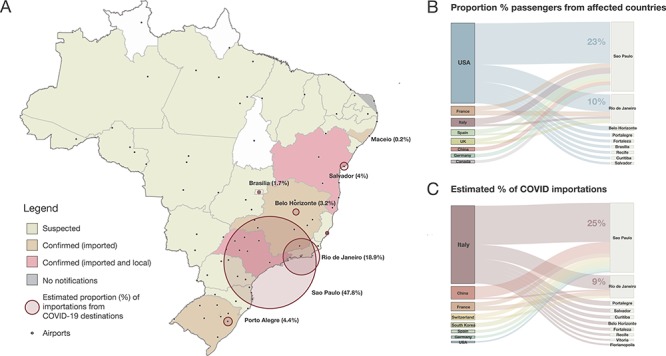
Potential for COVID-19 importation in Brazil. (A) Map of Brazilian federal states and federal district coloured according to COVID-19 notification status (as of 10 March 2020). Circles correspond to the estimated proportion of arrivals from the top 29 destinations (except Iran and Portugal) that had reported local COVID-19 by 5 March 2020. (B) Percentage of passengers for the top-20 routes to Brazilian airports from countries that had reported COVID-19 cases by 5 March 2020. (C) Estimated percentage of importations for the top-20 routes from countries that had reported local COVID-19 by 5 March 2020.

To better understand the potential for SARS-CoV-2 introductions to Brazil, we estimate the relative risk of COVID-19 introduction to Brazilian cities by taking into account SARS-CoV-2 incidence per international traveller arriving at an airport in Brazil. We estimate that 54.8% of all imported cases would be expected to come from travellers infected in Italy and 9.3% and 8.3% of the cases would be from travellers infected in China and France, respectively. The route Italy–São Paulo was estimated to comprise 24.9% of total infected travellers flying to Brazil during this period. Moreover, we estimate that Italy has been the source location for five of the top 10 importation routes for infected travellers into Brazil based on the current epidemiological scenario (Supplementary data). Consistent with this, at least 48% (*n* = 14/29) of the reported imported cases in Brazil have a history of travelling to Italy prior to onset of symptoms, as of 9 March 2020. Six (23.1%) of the confirmed cases that acquired the virus in Italy have been identified in São Paulo (Supplementary data).

We found that the proportion of estimated imported cases by city of destination is highly correlated with the proportion of detected imported cases. Our study has several limitations. Unfortunately, data from Iran and Portugal were not available for this analysis. Moreover, our analysis relies on incidence data, and thus, the risk of importation will follow changes in epidemic sizes at source locations. In fact, with the reduction in the number of flights leaving from Italy and 51% of flights to Brazil departing from airports in the USA, we should anticipate an increasing proportion of infected travellers arriving from the USA. Moreover, the estimated risk of importation from China is likely an overestimate as recent measures have extensively decreased the flights to Brazil.

At a time when the number of SARS-CoV-2 cases is steadily growing in Brazil, our findings highlight the high potential for the introduction of new cases in several cities of Brazil, especially in São Paulo and Rio de Janeiro metropolises. Rapid identification of locations where clusters of local transmission might first ignite is critical to better coordinate preparedness, readiness and response actions.^3^^,^^4^ There is a critical need for epidemiological, human mobility and genetic data^5^ to understand virus transmission dynamics at local, regional and global scales. Continued integration of these data streams should help guide the deployment of resources to mitigate COVID-19 transmission.

## Funding

This work was supported by a Medical Research Council and Fundação de Amparo à Pesquisa do Estado de São Paulo CADDE partnership award (MR/S0195/1) and a John Fell Research Fund (grant 005166). N.R.F. is supported by a Sir Henry Dale Fellowship (204311/Z/16/Z). D.D.S.C. is supported by the Clarendon Fund and by the Oxford University Zoology Department.

## Authors statements

K.K. is the Founder of BlueDot, a social enterprise that develops digital technologies for public health. K.K. and A.W. are employed at BlueDot. D.S.C., L.A., M.K., W.O., J.C., E.C.S., O.G.P. and N.R.F. have no conflicts of interest to declare.

## Authors contributions

D.S.C., L.A. and N.R.F. conceived the idea and wrote the manuscript. D.S.C., L.A., N.R.F., K.K. and A.W. conducted data analysis. D.S.C., N.R.F., L.A., M.U.G.K., W.O., J.C., E.C.S., O.G.P., A.W. and K.K. interpreted data and contributed to writing.

## Supplementary Material

Candido_SM_taaa042
