# Supplementary material for: Routes for COVID-19 importation in Brazil
Source: J Travel Med. 2020 Mar 23;27(3):taaa042. doi: 10.1093/jtm/taaa042 (PMC7184379; doi:10.1093/jtm/taaa042)
Supplement: Candido_SM_taaa042 [file candido_sm_taaa042.docx]

***Supplementary Information***

**Routes for COVID-19 importation in Brazil**

**Running title: COVID-19 importation in Brazil**

Darlan Da S Candido, MSc^1^, Alexander Watts, PhD ^2,3^, Leandro Abade, DPhil ^1^, Moritz UG Kraemer, DPhil ^1,4,5^, Prof Oliver G Pybus, DPhil ^1,6^, Prof Julio Croda, MD, PhD ^7,8,9^, Wanderson de Oliveira, PhD ^7^, Kamran Khan, MD, MPH ^2,3^, Prof Ester C Sabino, PhD ^10^, Prof Nuno R Faria, PhD^1,10^

Correspondence to Nuno Rodrigues Faria ([nuno.faria@zoo.ox.ac.uk](mailto:nuno.faria@zoo.ox.ac.uk))

***Historical air travel data***

Historical air travel data was obtained for 29 of the countries with the highest number of SARS-Cov-2 cases as of 5^th^ March 2010 (see list below). Data from the International Air Transport Association (IATA) was used to estimate the number of international travellers departing from each of the 29 countries and having Brazil as a final destination. IATA data corresponds to 90% of all worldwide trips on commercial flights from February to March 2019, and market intelligence was used to model the remaining data for the same period^1^. No information on possibly interrupted journeys was available.

***SARS-CoV-2 Incidence***

SARS-CoV-2 incidence for each of the 29 of the countries was calculated using the confirmed SARS-CoV-2 number of cases reported by World Health Organization as of 9^th^ March 2020^2^ and the total population for each country for 2019 from the United Nations World Population Prospects 2019 database {UN, 2019 #4876}. A total of 29 countries used in this study: Algeria, Australia, Canada, China, Croatia, Denmark, Ecuador, Finland, France, Germany, Greece, Indonesia, Israel, Italy, Japan, Lebanon, Malaysia, Netherlands, Norway, Singapore, South Korea, Spain, Sweden, Switzerland, Thailand, United Arab Emirates, United Kingdom, United States of America, Viet Nam.

***SARS-CoV-2 importation estimates***

Estimates on the proportion of expected importations (E) for each air travel route were calculated using the incidence for each route (i) and the number of passengers (p) (historical air travel data) following $E=i*p/\sum i$ *100. The expected proportion of importations per country of origin (e.g. Italy) was calculated as the sum of (E) for all routes for starting at that specific country. Finally, the expected proportion of importations per Brazilian destination (e.g. Sao Paulo) was estimated as the sum of (E) for all routes ending at that specific destination, regardless of the country of origin.

***Correlation between estimation and cases***

To assess the accuracy of our estimates, we ran a correlation analysis between the estimated number of imported cases per final destination in Brazil (e.g. Sao Paulo) and the actual number of imported cases as reported by the Brazilian Ministry of Health on the 9^th^ of March 2020. We fitted a simple linear regression model using RStudio Version 1.2.1335.


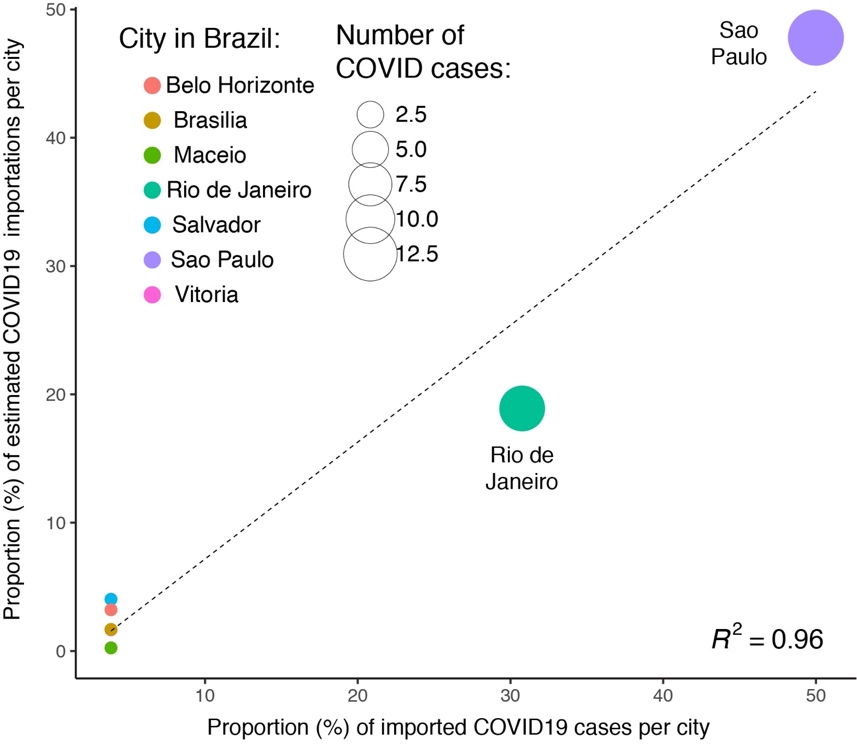


**Figure S1**. Correlation between the estimated proportion of imported COVID-19 cases per Brazilian city and the proportion of COVID importations reported Brazilian Ministry of Health (as of 9^th^ March 2020). Circles are coloured according to location in Brazil and sized according to the number of COVID-19 imported cases reported Brazilian Ministry of Health (as of 9^th^ March 2020). A linear regression model was fitted to the data using RStudio Version 1.2.1335.

**References**

1. Passenger Intelligence Services (PaxIS). Montreal: International Air Transport Association; 2017. Available from http://www.iata.org/services/statistics/intelligence/paxis/Pages/index.aspx [cited 2018 Jun 11].

2. WHO. Coronavirus disease (COVID-2019) situation reports. <https://www.who.int/emergencies/diseases/novel-coronavirus-2019/situation-reports>; 2020.
